# OPTIMIZATION OF WEB-BASED COGNITIVE BEHAVIORAL THERAPY FOR INSOMNIA NITECAPP IN RURAL DEMENTIA CAREGIVERS

**DOI:** 10.1093/geroni/igae098.0135

**Published:** 2024-12-31

**Authors:** Christina McCrae

**Affiliations:** University of South Florida, Tampa, Florida, United States

## Abstract

Informal dementia caregivers (CGs) often experience chronic insomnia that contributes to stress and poor health. Rural CGs are particularly vulnerable but have limited access to CBT for insomnia (CBT-I), a recommended treatment for chronic insomnia. Web delivery holds promise for improving outcomes and access to this frontline treatment. Here we describe the iterative development of web CBT-I for rural dementia CGs. Qualitative and quantitative evaluations were conducted to explore user needs and adapt/validate content using a stepwise approach: 1- advisory panel of rural CGs (n=5) and primary care providers (PCPs; n=5) gave feedback used to adapt NiteCAPP, 2-focus groups of CGs (n=5) and PCPs (n=7) reviewed the newly adapted intervention and gave additional feedback, 3-NiteCAPP CARES was piloted in CGs (n=5) to test feasibility and establish preliminary effects. Fourteen daily electronic sleep diaries and questionnaires assessing arousal, health, mood, burden, subjective cognition, and interpersonal processes were collected pre/post treatment. Feedback recommended streamlining content, eliminating jargon, and including CG focused content. Multiple features were evaluated positively (comprehensive and engaging information, CG focus, layout, easy-to-access material, easy-to-understand sleep graphs). Suggestions for improvement included giving day/night viewing options, collapsible text, font size options, tabbed video access, and a glossary. Preliminary effects on CG sleep, arousal, health, mood, burden, cognition, and interpersonal processes (ps<.05) were promising. Targeted web CBT-I may increase access and improve CG outcomes. NiteCAPP CARES is now being evaluated in NIA-funded RCT.

